# Analecta

**Published:** 1842-04-09

**Authors:** 


### ANALECTA.

*Lithotrity in a Child.*—At a meeting of the Academy of Medicine, Paris, January 3d, M. Segelas presented a boy, 23 months old, whom he had successfully delivered of a stone, 14 lines in diameter, by the operation of lithotrity. This was the youngest individual on whom he had, as yet, operated. A great number of sittings was required, but no bad symptom occurred. M. Segalas brought forward this case in support of the opinion which he sustains against most other surgeons—viz., that lithotrity may and should be applied to children. The arrest of the fragments of calculus by the narrow uretha of the child does not seem to him to be a valid objection, because the fragments may be broken up in the canal.

*Extirpation of Uterine Polypi.*—At the meeting of January 18th, M. A. Berard, who at a previous meeting, had shown a fibrous polypus which he had removed from the cavity of the uterus, after dividing the neck of that organ, exhibited another fibrous tumour, which he had extracted in the same manner, and with equal success. The patient had laboured for a considerable time, under uterine hæmorrhage, which had reduced her to the lowest state of exhaustion. Although the orifice of the uterus was open, it was not sufficiently so to allow the tumour to pass through it. M. Berard divided with a bistoury both sides of the os tincæ, and the tumour immediately descended into the vagina. The operator now fixed a hook into it, drew it down to the vulva, and removed it by cutting across the pedicle with a scissors. The uterus ascended quickly to its natural position, and the hæmorrhage did not recur. M. Berard thinks that facts of this kind should be made extensively known, for as many surgeons still think that a polypus enclosed in the uterine cavity is beyond the reach of operation, many females are allowed to perish whose lives might be saved by this method.—*Prov. Med. and Surg. Journ.*, Jan. 29, 1842

*On the treatment of Paraphymosis.* By J. Togood, Esq., of Bridgwater.—The incision of the stricture in Paraphymosis, is pretty generally practised by surgeons, except when this disease occurs in children, and in such the reduction is often effected by pressure. I have seen repeated incisions fail of relieving the stricture, and leave foul and intractable sores, which have been extremely tedious and difficult to heal. I have always succeeded in reducing a paraphymosis, either in an adult or child, however long standing, without having recourse to the knife, by the following method:—I place the patient against a wall, and take care that he is steadily supported by an assistant on each side; a piece of linen cloth is then laid over the glans, and with my thumbs I knead the blood out of it, drawing the prepuce forwards at the same time with two fore fingers of each hand. A steady perseverance in this plan never fails, and, although the operation is a painful one, the patient is amply rewarded by the rapidity of the cure, which requires nothing more than the application of a saturnine lotion for two or three days.—*Ibid. Jan.* 15, 1842.

---

*Idiopathic Hydrophobia.* By John Kimbell, M. R. C. S. L.—W. K., aged 24 years, of a bilious and lymphatic temperament, has, during the last month, suffered from occasional attacks of palpitation of the heart, occurring generally in the night, and invariably followed by profuse perspiration. On October 4, 1841, he rode a distance of fourteen miles, and, on arriving at the end of his journey, about twelve o'clock, A. M., he was seized suddenly with great difficulty of breathing, pain over the region of the heart, and painful sensations over the chest. The paroxysm continued for a few minutes, when the dyspnœa and pain gradually subsided; he afterwards ate a good dinner, and appeared as well as usual, until about eight o'clock in the evening, when all the symptoms returned with greater violence than before, and to so distressing a degree did the dyspnœa increase, that there appeared to be imminent danger of suffocation. He was now bled to eighteen ounces, but without any manifest relief, and the operation was repeated in three hours to the amount of six ounces, which had the effect of considerably relieving the pain. About 5, A. M., Oct. 5, I saw him; he could not speak, although conscious of what was passing around him; I was informed that he had had violent convulsive movements of the arms, which had lasted nearly an hour, and he now appeared to be suffering from a spasmodic constriction about the glottis and pharynx, causing extreme difficulty of inspiration, which had a peculiar crowing character; he had likewise a great desire for water, and complained much of thirst; no sooner, however, was this fluid brought into his presence, than it was obliged to be withdrawn; the sight of it caused an alarming increase of pain about the larynx with a horrible feeling of suffocation, but with the removal of the water the symptoms became ameliorated. From so many hydrophobic symptoms being present, I was apprehensive he might have been bitten by a dog, and questioned him upon the subject very closely, but to all my interrogations he shook his head negatively. During the intervals of ease, his pulse was full and soft, and averaged eighty beats in a minute; his tongue was clean; the bowels were regular; and the skin of the natural temperature. Aware that there was predisposition to spinal disease, I examined the back, and found about the lower part of the cervical region, tenderness upon pressure, and I observed that this *pressure invariably* produced an exacerbation in all the symptoms, and of this I fully satisfied myself, and my patient likewise, by repeating the pressure three or four times. A blister was applied over this spot; it rose well, and he soon became able to swallow. Doses of opium were given by the mouth, and an opium injection was administered *per rectum.* I should have stated that, from the commencement of the attack up to the present period, he has experienced a great difficulty in passing his urine, but none in voiding his fæces. 5th. Much improved in every respect, but when his head was raised the spasm was speedily reproduced. He had a constant smacking of his lips, and frequent twitches in his legs and feet; the right arm found partially paralysed. No headache; no confusion of intellect. 7th. Still improving.—Spasms had entirely disappeared; he could swallow fluids with the greatest ease; tongue clean, bowels well opened, secretions healthy; he can now be raised without suffering. The blister discharged freely. The dorsal region was rubbed with an embrocation containing croton oil, tartar emetic, &c., and quinine was given during the day, with henbane at night. From this period he gradually progressed, and at the end of the month was thought sufficiently improved to resume his avocation. One day, however, previous to his intended departure, he had a return of the dyspnœa, but in a much less degree than before. This was immediately treated with the application of leeches to the cervical region, followed by a blister, when all the symptoms soon vanished. He has two issues, one on each side of the cervical vertebræ, which discharged freely, and he may now be considered convalescent.—*Ibid. Jan.* 8, 1842.

---

*Hepatic Congestion: diagnosis; treatment.* By C. J. B. Williams, M. D., F. R. S, Professor of the Practice of Medicine and of Clinical Medicine.—The next cases to which I would direct your attention, are those of Mary Hennen, æt. 20, and Mary Hill, æt. 37; both cases of disordered digestion, with congestion of the liver. Referring you to the books for a very full report of their cases, I may remind you that the former was admitted Oct. 26th, complaining of pain in the epigastrium not increased by pressure, and frequent vomiting, so that she could retain no food on her stomach.—No heat of skin; tongue slightly furred, with red spots. These symptoms were at once relieved by a blister to the epigastrium, and a draught with hydrocyanic acid and carbonate of soda. Blue pill at night, and castor oil in the morning, were also given.

Nov. 1st, was nearly well; but on the 2d complained of more pain in the right hypochondrium. The dull stroke sound of this region extended not lower, but higher, than usual; to the breast in front, and nearly to the lower edge of the scapula behind. Was this dulness from enlarged liver, or from effusion in the right pleura? We found that the breath sound was heard pretty low down into the dull region, without rhonchus, and was here sharper than usual; but without bronchial breath or voice sound above. On trying degrees of percussion in the manner which I have often recommended to you, we readily solved the question: gentle percussion gave a more hollow sound than strong percussion; showing that the lung, the seat of the breath sound, was superficial; and the denser body giving the duller sound on strong percussion, was deeper seated, and must therefore be the liver. This organ was, therefore, enlarged; and the absence of the active symptoms of hepatitis left to us the conclusion that the enlargement was congestive; a species of swelling to which the venous constitution of the liver renders it especially liable. As causing this congestion, we might refer to a cold, with headache, drowsiness, &c., caught about three weeks before admission; but we must also take into account the fact, that this girl, although not deficient in blood, had not menstruated for five months. We had assured ourselves, by the pallid nipples and the absence of tumor or uterine murmur in the abdomen, that pregnancy was not the cause of the amenorrhœa.

The treatment employed to remove the congestion of the liver, was by means calculated to increase its secretion, repeated doses of calomel and blue pill, with a little antimonial powder, a daily dose of castor oil, and a blister to the side. Under these means the pain in the side soon left her; and on the 9th the dulness of the right side was nearly gone. She was given compound decoction of aloes twice a day to keep open the bowels, and to promote the return of the catamenia. She will be liable to a return of her disorder until the periodic relief is restored; but as that is not more likely to take place in the hospital than out of it, she was discharged apparently well.

The other patient, Mary Hill, had long been subject to bilious attacks, with sickness, costiveness, and pain in right side. The present attack began six weeks before her admission (Nov. 6) with severe pain in the abdomen, and vomiting, for which she was treated by leeching, mustard poultices, aperients, &c., which relieved the pain in the abdomen, but left pain and tenderness in the right hypochondrium and scapula, sickness, and headache. There was dulness in the lower part of the right chest, chiefly at the side and back. Tongue clean; thirst; pulse natural; bowels not open three days. CC. Hypochon. dextro ad ℥viij. ℞ Hydrarg. Chlorid. Pulv. Antimonialis, aa. gr. ij. Pil. Hydrarg. Extr. Conii, aa. gr. iij. Fiant pilulæ ij. bis die sumendæ. ℞ Sodæ Tart. ʒij. Sodæ Sesquicarb. ʒss. Aquæ Menthæ, Aq. Font. aa. ℥i. Fiat haustus bis die sumendus.

These medicines were continued three days, and with the aid of senna draught opened the bowels freely, and relieved the symptoms, except the pain in the right side. On the 13th, the catamenia appeared; all medicines were suspended until the 16th, when the pain and dulness were almost gone, and left entirely after an aperient. She was discharged well on the 19th.

This case illustrates the influence of the natural periodic discharge in assisting to dissipate congestion of the liver; and I would remind you, that this natural means of relief is, in slight cases, for many reasons, better than the continued use of medicine.—*Lon. Med. Gaz. Dec.* 3, 1841.

---

*Blistering for Warty Excrescences on the Skin.* By W. Davidson, M. D., Physician to the Glasgow Infirmary.—Neil M'Kinnon, plasterer, aged thirty-five, admitted 14th December, 1840. Left lower extremity was twice as thick as right; cellular texture presented the appearance, and gave the sensation of being much hypertrophied, offered much resistance to the fingers when pressure was made, and only pitted slightly. The ham and the two upper and posterior thirds of leg were covered with deep rather fleshy warty excrescences, traversed by deep irregular longitudinal fissures; but in the popliteal space they were transverse. A patch of a similar nature, but dark-coloured, and resembling icthyosis, about the size of the hand, existed over the lower part of the leg at flexion of the ankle joint.

The colour of the patches was generally brownish red. Over anterior part of same leg there were numerous pretty large yellow scabs, and the skin covering the whole of inner and upper part of thigh, was of a dark livid colour, as was also a patch over inner part of right thigh, which, at one period of the disease, was also encrusted with scabs. The patient ascribed the affection to a fall. It made its appearance in the form of warts on the lower and inner third of the leg about five years ago. He stated that he was cured in this Hospital about two years ago, but that the limb retained its blue appearance, which he says was always the precursor of the warty excrescences. His general health was good, pulse and bowels regular, tongue clean. App. sol. arsenical. part. affect. quotidie. Cap. pil. 1 colocynth. comp. omni nocte.

28th *Dec.*—Warty excrescences are in much the same state. ℞. Chlor. zinci ʒj. Aquæ ℥ij. Solve. App. sol. part. verrucos. Cap. T. Canth. gtt. xx. ter in die.

The strength of the solution of chloride of zinc was gradually increased, until it amounted to three drachms of this salt to an ounce of water, on the 20th January, 1841, while he had been taking an arsenical solution internally from the 11th of the same month; but there was only a slight improvement, there being still an enormous thickness of warty structure. On the 22nd January, a blister was ordered to a portion of the leg, which acted well, and produced a large detachment of warty substance. The blisters were repeated every two or three days until the whole affected surface had been more than once vesicated. Severe strangury was several times produced, even by the tela vesicatoria, which was employed on two or three occasions in this case.

On the 14th February he was dismissed, the right leg appearing to be quite free from warty excrescences, and there remaining on the left only a slight thickening of the skin near the ankle, where the disease resembled 'cthyosis.—*Lond. & Ed. Journ. of Med. Science.*

---

*A Medical Board* has been ordered to convene in Philadelphia, on the 2d of May next, for the examination of Assistant Surgeons for promotion, and of candidates for appointment in the Medical Staff of the Army. The Board is composed of Surgeon T. G. Power, President; Surgeon H. A. Stinnecke and Assistant Surgeon J. M. Cuyler, Members.

